# Nutritional management of phenylalanine hydroxylase (PAH) deficiency in pediatric patients in Canada: a survey of dietitians’ current practices

**DOI:** 10.1186/s13023-018-0978-0

**Published:** 2019-01-08

**Authors:** Nataliya Yuskiv, Beth K. Potter, Sylvia Stockler, Keiko Ueda, Alette Giezen, Barbara Cheng, Erica Langley, Suzanne Ratko, Valerie Austin, Maggie Chapman, Pranesh Chakraborty, Jean Paul Collet, Amy Pender, In collaboration with the Canadian Inherited Metabolic Diseases Research Network (CIMDRN)

**Affiliations:** 1 University of British Columbia, Vancouver, British Columbia Canada; 2 University of Ottawa, Ottawa, Ontario Canada; 3 British Columbia Children’s Hospital, Vancouver, British Columbia Canada; 4 Children’s Hospital of Eastern Ontario, Ottawa, Ontario Canada; 5 Children’s Hospital of Western Ontario, London, Ontario Canada; 6 The Hospital for Sick Children (SickKids), Toronto, Ontario Canada; 7 IWK Health Centre Medical Genetics, Halifax, Nova Scotia Canada; 8 McMaster Children’s hospital, Hamilton, Ontario Canada

**Keywords:** PAH deficiency, Phenylketonuria, PKU, Management practices, Nutrition management, PAH deficiency practice guidelines, Metabolic dietitians’ survey

## Abstract

**Background:**

Phenylalanine hydroxylase (PAH) deficiency is one of 31 targeted inherited metabolic diseases (IMD) for the Canadian Inherited Metabolic Diseases Research Network (CIMDRN). Early diagnosis and initiation of treatment through newborn screening has gradually shifted treatment goals from the prevention of disabling complications to the optimization of long term outcomes. However, clinical evidence demonstrates that subtle suboptimal neurocognitive outcomes are present in the early and continuously diet-treated population with PAH deficiency. This may be attributed to variation in blood phenylalanine levels to outside treatment range and this, in turn, is possibly due to a combination of factors; disease severity, dietary noncompliance and differences in practice related to the management of PAH deficiency. One of CIMDRN’s goals is to understand current practices in the diagnosis and management of PAH deficiency in the pediatric population, from the perspective of both health care providers and patients/families.

**Objectives:**

We investigated Canadian metabolic dietitians’ perspectives on the nutritional management of children with PAH deficiency, awareness of recently published North American treatment and nutritional guidelines in relation to PAH deficiency, and nutritional care practices within and outside these guidelines.

**Methods:**

We invited 33 dietitians to participate in a survey, to ascertain their use of recently published guidelines and their practices in relation to the nutritional care of pediatric patients with PAH deficiency.

**Results:**

We received 19 responses (59% response rate). All participants reported awareness of published guidelines for managing PAH deficiency. To classify disease severity, 89% of dietitians reported using pre-treatment blood phenylalanine (Phe) levels, alone or in combination with other factors. 74% of dietitians reported using blood Phe levels ≥360 μmol/L (6 mg/dL) as the criterion for initiating a Phe-restricted diet. All respondents considered 120-360 μmol/L (2–6 mg/dL) as the optimal treatment range for blood Phe in children 0–9 years old, but there was less agreement on blood Phe targets for older children. Most dietitians reported similar approaches to diet assessment and counseling: monitoring growth trends, use of 3 day diet records for intake analysis, individualization of diet goals, counseling patients to count grams of dietary natural protein or milligrams of dietary Phe, and monitoring blood Phe, tyrosine and ferritin.

**Conclusion:**

While Canadian dietitians’ practices in managing pediatric PAH deficiency are generally aligned with those of the American College of Medical Genetics and Genomics (ACMG), and with the associated treatment and nutritional guidelines from Genetic Metabolic Dietitians International (GMDI), variation in many aspects of care reflects ongoing uncertainty and a need for robust evidence.

## Background

Phenylketonuria (PKU; OMIM 262600) is an autosomal recessive inborn error of phenylalanine metabolism caused by a deficiency of the phenylalanine hydroxylase (PAH) enzyme. PAH deficiency encompasses a spectrum of biochemical phenotypes from classic PKU (severe PAH deficiency) to mild hyperphenylalaninemia (with varying degrees of residual PAH activity). Untreated, PAH deficiency is characterized by elevated phenylalanine (Phe) levels in the blood and brain, resulting in neurological damage via impaired neurotransmitter metabolism and direct phenylalanine neurotoxicity [1]. Ground-breaking universal newborn screening for PAH deficiency, and treatment with a Phe-restricted diet and Phe-free or low Phe medical foods (formulas), have virtually eliminated severe PAH deficiency-related complications in early and continuously treated individuals in many populations throughout the world. This important achievement has shifted the goals of treatment from prevention of profound intellectual disability to optimization of health outcomes. Nutrition therapy, which aims at both maintaining blood Phe concentrations within treatment goals and meeting individual nutritional needs, remains a cornerstone of the management of PAH deficiency [1–4]. If administered appropriately and adhered to consistently, currently available treatment modalities are expected to result in health outcomes comparable with the general population. However, despite the medical and public health success story of the treatment of PAH deficiency, evidence suggests that long-term patient outcomes are not always optimal. Individuals living with PAH deficiency have higher risks of deficits in neurocognitive domains such as working memory, attention, processing speed and motor control, behavioural and psychosocial issues, growth and nutritional deficiencies, brain and bone pathology, and quality of life [5–8]. Delayed age at initiation of therapy, as well as variable lifelong blood Phe levels and nonadherence to treatment, have been identified as major contributors to the development of suboptimal outcomes [5, 9]. It has been argued that delivery of health care that is not aligned with established best practice, uncertainty in clinical decision making, and inconsistent access to care may also contribute to suboptimal outcomes for some patients [7, 10, 11].

Relatively robust published evidence exists to support recommendations for many areas of management of PAH deficiency, such as diagnosis, treatment onset and duration, therapeutic goals, treatment targets and organization of care [12, 13]. However, as with other rare diseases, high quality empirical evidence is not always available to support treatment decisions, resulting in several areas of uncertainty and inconsistencies in clinical decision-making that may ultimately lead to variability in health outcomes. For example, it is commonly agreed that life-long nutrition treatment should start as soon as possible for infants with initial untreated blood Phe levels > 600 μmol/L (10 mg/dL) [3]. However, the evidence regarding the possible beneficial effect of a life-long Phe-restrictive diet in children whose initial untreated blood Phe levels are 360–600 μmol/L (6–10 mg/dL) is sparse, leading to rather provisional recommendations for this patient subgroup [3, 14]. Variability in the initiation of diet therapy, and other management practices related to PAH deficiency, have been reported both across countries and across centres within the same countries [1, 15]. This may reflect in part the differences in treatment guidelines for PAH deficiency, developed by different groups and in different jurisdictions [1, 15].

The lack of uniformity in the management of PAH deficiency and new published evidence prompted the development of updated broad-based clinical guidelines (published by the American College of Medical Genetics and Genomics, ACMG) [3] and companion recommendations for the nutritional management of PAH deficiency (published by Genetic Metabolic Dietitians International, GMDI) [2], with the goal of improved patient care in North America. Both guidelines relied on independent evidence reviews conducted by experts from the National Institutes of Health and Agency for Healthcare Research and Quality [16, 17]. Both guidelines also integrated this evidence with a consensus of expert opinion in clinical practice areas for which evidence was lacking. For example, the development process for the nutritional management guidelines from GMDI included published evidence reviews, clinical protocols, consensus of experts via Delphi surveys and nominal group expert meetings, an external review, field testing, and revision, to reach at least 75% agreement [18]. The recent publication of these guidelines, coupled with previous research documenting variation in care, presented an opportunity to investigate how the guidelines are perceived by Canadian healthcare providers, and to identify important variations in care.

In this study, we aimed to ascertain Canadian metabolic dietitians’ awareness of published guidelines for PAH deficiency and their approaches to nutritional management of PAH deficiency in the pediatric population. Identifying uncertainties in the nutritional management of pediatric PAH deficiency in Canada, from practitioners’ perspectives, is important for understanding the impact and uptake of the new guidelines, identifying areas where knowledge translation and mobilization are needed, and prioritizing questions about treatment effectiveness for future research. This survey was distributed in 2016, and thus our primary comparison was with the recently published North American management guidelines for PAH deficiency [2, 3].

## Materials and methods

### Questionnaire

Notwithstanding the challenges of management of adult phenylketonuria (PKU), especially of maternal PKU, we developed the survey with the pediatric population in mind. Many treatment concerns are different, and our focus on pediatric phenylalanine hydroxylase (PAH) deficiency is consistent with one of the goals of the Canadian Inherited Metabolic Diseases Research Network (CIMDRN); to understand current practices in the diagnosis and management of PAH deficiency in the pediatric population, from the perspective of both health care providers and patients/families.

The team of investigators included experienced registered metabolic dietitians from several Canadian metabolic centres, as well as a metabolic physician and investigators with expertise in survey research methods. We developed a study-specific questionnaire with 52 questions that covered self-reported awareness and use of the most recent published North American guidelines, personal and practice characteristics, and the following topics related to the nutritional management of PAH deficiency: classification of disease severity; frequency of monitoring and target ranges for surrogate biomarkers; recommended dietary intakes of key nutrients and methods recommended for patients to self-monitor intake of these nutrients; recommended use and accessibility of medical foods (formulas); use of vitamin/mineral supplements; frequency of clinic visits and communication with patients and their families; and methods of encouraging and monitoring patient adherence to therapy.

The survey questionnaire is available as a supplementary material.

### Sample selection and survey implementation

Eligible participants were metabolic dietitians who provided care to children with PAH deficiency in Canada. Based on their clinic listing on the Genetic Metabolic Dietitians International (GMDI) website, we identified 33 Canadian metabolic dietitians in nine Canadian provinces and three territories. We could not be certain, based on the available information, that these dietitians specifically provided care to children with PAH deficiency; this eligibility criterion was thus incorporated into the questionnaire as a screening question.

Adapting Dillman’s tailored design method [19], we made up to six contacts (between March and May 2016) to invite Canadian metabolic dietitians to participate in the survey. These included (a) a pre-notification email message sent out by one of the study investigators who is a metabolic dietitian; (b) an initial mailed invitation with a copy of the survey; (c) an initial email invitation with the link to the online survey; (d) a mailed reminder letter with a copy of the questionnaire, sent to non-respondents; (e) an email-reminder with the link; and (f) a final reminder email message, sent to remaining non-respondents.

Dietitians could respond to the survey by mail, using a prepaid return envelope that was included with each of the two questionnaires that were mailed; or online, through a REDCap platform, hosted on a secure BC Children’s Research Institute server with participant access through a unique identification number and password.

In accordance with existing evidence regarding monetary incentives [20], we offered a $25 iTunes gift card to each participant who completed the survey; and this was mentioned in the invitation letters and subsequent reminders.

### Analysis of survey data

Data were entered into a REDCap database and exported to SAS^®^ 9.4 Software for descriptive analysis. We report proportions as all survey questions were categorical. Many questions used 4 or 5-point Likert-type scales and single-answer option; alternatively, some questions incorporated multiple answers which were expected to add up to more than 100%. Where necessary and applicable, we grouped categories (e.g., “all” with “most”, “excellent” with “good”, “sometimes” with “rarely”) to account for small numbers.

## Results

### Response rate and distribution of sample characteristics

Of 33 Canadian metabolic dietitians invited to participate, we received twenty surveys of which nineteen had been completed. One respondent indicated on an initial screening question that he/she did not provide care for pediatric phenylketonuria (PKU), and therefore did not complete the full questionnaire (response rate, 19/32, 59%). Ten surveys (53%) were submitted on paper and 9 (47%) were completed online. We received responses from 14 centres, located in nine of the ten Canadian provinces. Of the 14 centres, 10 had only one respondent and a further 4 centres had multiple respondents. The majority of respondents had worked in metabolic nutrition services for more than 6 years (74%), were full time (68%), and dedicated at least half of their time to the care of children with phenylalanine hydroxylase (PAH) deficiency (53%) (Table 1). At the centre level: respondents indicated that the majority of centres (79%) followed more than 20 patients with PAH deficiency who required regular nutrition services; and cared for both pediatric and adult populations (79%). Only three centres (21%) were reported to have a comprehensive multidisciplinary team that includes a metabolic physician, metabolic dietitian, metabolic nurse, psychologist, social worker and clinical biochemist (Table 1).

**Table 1:** Sample Characteristics

Participant Characteristics	Participant n (%)
Total number of years of practice in nutrition services (*n* = 19 dietitians)	
< 1 year	2 (11%)
1–2 years	1 (5%)
3–5 years	2 (11%)
6–10 years	5 (26%)
11–15 years	4 (21%)
> 16 years	5 (26%)
Working full-time (*n =* 19 dietitians)	13 (68%)
Working part-time	6 (32%)
Time dedicated to care of PAH deficiency patients (*n =* 19 dietitians)	
All of my time	3 (16%)
Less than all of my time but at least half of my time	7 (37%)
Less than half of my time	9 (47%)
Centre Characteristics	Centre n (%)
Number of PAH deficiency children actively followed at centre (*n* = 14 centres)	
< 10	1 (7%)
10–20	2 (14%)
20–40	8 (57%)
> 40	3 (22%)
# PAH deficiency patients newly diagnosed each year (*n =* 14 centres)	
2 or fewer	12 (86%)
3–5	2 (14%)
PAH deficiency patient population treated at the centre (*n =* 14 centres)	
Pediatric only (0–18 years)	3 (21%)
Pediatric and adult combined	11 (79%)
Centre’s health care team composition (*n =* 14 centres)^a^	
Dietitian	14 (100%)
Physician	13 (93%)
Metabolic nurse	9 (64%)
Social worker	7 (50%)
Psychologist	6 (43%)
Clinical biochemist	5 (36%)
Genetic counselor	4 (29%)
Nurse-practitioner	1 (7%)
Units used: mg/dL^b^ (*n =* 14 centres)	2 (14%)^b^
Units used: μmol/L	12 (86%)

^a^Multiple choice question: the percentages are expected to add up to more than 100%

^b^One of the two centers reporting using mg/dL, uses mg/dL in older patients and umol/L in younger patients

### Use of published management guidelines of PAH deficiency (PKU)

All respondents were aware of published PKU guidelines, referencing the ACMG PKU consensus guideline^3^ and the companion recommendations for the nutrition management of PAH deficiency [2]. Other guidelines that participants mentioned included: “SERC-GMDI PKU Nutrition Management Guidelines” [21], “NIH Consensus Guideline for Management of PKU” [16], “European Guidelines (not specified)”, “Publications by Anita Macdonald (not specified)” and “Nutrition Management of Inherited Metabolic Diseases” [22].

### Opinions on classification of PAH deficiency severity

**Fig. 1:**
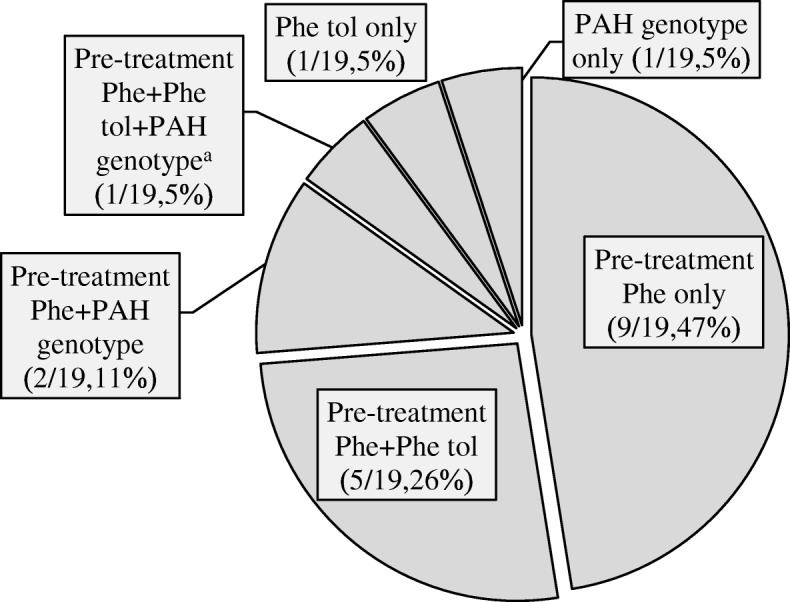
^a^The respondent also indicated using Phe blood levels when the patient is catabolic

To classify the severity of PAH deficiency 9 of 19 respondents (47%) reported using only newborn pre-treatment blood phenylalanine (Phe) levels and 8/19 (42%) used pre-treatment blood Phe levels in combination with either Phe tolerance, PAH genotype or all three (Fig. 1). One respondent also indicated using blood Phe levels when the patient is catabolic.

We also asked respondents to indicate the specific pre-treatment blood Phe levels that they used to categorize PAH deficiency severity, using typical classification terminology of classical, moderate, and mild PKU, and mild HPA [23] (Table 2). Definitions of these categories varied among respondents.

**Table 2:** Reported definitions of PAH deficiency phenotype based on pre-treatment Phe levels^a^

Reported Phe ranges	N of respondents/total N of responded dietitians
How do you define “Mild HPA” based on blood Phe levels in the newborn before treatment?	
< =360 μmol/L (<=6 mg/dL)	1/9
< 360 μmol/L (< 6 mg/dL)	3/9
120–360 μmol/L (2–6 mg/dL)	2/9
200–360 μmol/L (3.33–6 mg/dL)	1/9
360–600 μmol/L(6–10 mg/dL)	1/9
< 600 μmol/L (< 10 mg/dL)	1/9
How do you define “Mild PKU” based on blood Phe levels in the newborn before treatment?	
120–360 μmol/L (2–6 mg/dL)	1/9
360–600 μmol/L (6–10 mg/dL)	3/9
< 600 μmol/L (< 10 mg/dL)	1/9
600–900 μmol/L (10–15 mg/dL)	2/9
360–1200 μmol/L (6–20 mg/dL)	2/9
How do you define “Moderate PKU” based on blood Phe levels in the newborn before treatment?	
> 360 μmol/L (> 6 mg/dL)	1/8
600–1000 μmol/L (10–16.67 mg/dL)	1/8
600–1200 μmol/L (10–20 mg/dL)	3/8
900–1200 μmol/L (15–20 mg/dL)	2/8
1200–1800 μmol/L (20–30 mg/dL)	1/8
How do you define “Classical PKU” based on blood Phe levels in the newborn before treatment?^b^	
> 600 μmol/L (> 10 mg/dL)	1/12
≥ 1000 μmol/L (> 16.67 mg/dL)	1/12
> 1200 μmol/L (> 20 mg/dL)	7/12
> 1200–1500 μmol/L (20–25 mg/dL)	1/12
1200–2400 μmol/L (20–40 mg/dL)	1/12
> 1800 μmol/L (> 30 mg/dL)	1/12

^a^Four respondents indicated that they do not use PKU classification based on pre-treatment Phe levels;

^b^Two respondents utilize pre-treatment Phe as follows: PKU is diagnosed when pre-treatment Phe is ≥1200 μmol/L

(classical PKU, included in “classical PKU”) and HPA < 1200 μmol/L (not included in the table)

### Blood phenylalanine levels in management and monitoring of phenylalanine hydroxylase deficiency 

The majority of respondents (74%) reported that they initiate dietary treatment at blood Phe levels of ≥360 μmol/L (≥6 mg/dL), although some dietitians support initiating treatment at higher Phe levels (Table 3).

**Table 3:** Blood Phe levels in treating and monitoring children with PAH deficiency

	n (%)
Blood Phe level (consistently elevated) to prompt initiation of a Phe-restricted diet (*n* = 19)	
≥ 360 μmol/L (≥6 mg/dL)	14 (74)
360–420 μmol/L (6–7 mg/dL)	1 (5)
≥ 420 μmol/L (≥7 mg/dL)	1 (5)
≥ 480 μmol/L (≥8 mg/dL)	2 (10)
≥ 600 μmol/L (≥10 mg/dL)	1 (5)
Optimal target range of blood Phe, by age	
0–12 months of age (*n =* 19)	
120–360 μmol/L (2–6 mg/dL)	19 (100)
> 1–2 years of age (*n =* 19)	
120–360 μmol/L (2–6 mg/dL)	19 (100)
> 2–10 years of age (*n =* 19)	
120–360 μmol/L (2–6 mg/dL)	19 (100)
> 10–18 years of age (*n* = 18)	
120–360 μmol/L (2–6 mg/dL)	14 (78)
120–600 μmol/L (2–10 mg/dL)	2 (11)
320–600 μmol/L (5.33–10 mg/dL)	2 (11)
Lowest acceptable average level of blood Phe as a long-term treatment goal, by age	
0–12 months of age (*n =* 18)	
100 μmol/L	1 (6)
120 μmol/L	17 (94)
> 1–2 years of age (*n* = 17)	
120 μmol/L	16 (94)
150 μmol/L	1 (6)
> 2–10 years of age (*n =* 17)	
120 μmol/L	16 (94)
150 μmol/L	1 (6)
> 10–18 years of age (*n =* 18)	
100 μmol/L	1 (6)
120 μmol/L	16 (89)
200 μmol/L	1 (6)
Would you be comfortable with a patient’s steady Phe level of < 120 μmol/L (*n =* 19)	
Yes	6 (32) ^a^
No	13(68)
Recommend maintaining higher-end therapeutic range blood Phe and more liberal natural protein intake (*n =* 19)	
For most/nearly all patients	5 (26)
For some patients	9 (47)
Rarely/never	5 (26)
Recommend maintaining lower-end therapeutic range blood Phe levels and more restricted natural protein intake (*n =* 19)	
For most/nearly all patients	4 (21)
For some patients	4 (21)
Rarely/never	11 (58)

^a^If yes, participants were asked to explain – open-ended responses: “If levels were consistent and testing was done weekly, I would be ok it with somewhat lower levels, perhaps as low as 80; I would be more comfortable with an older child (> 2 years), but this rarely happens; On Kuvan & hard to increase Phe intake; On restricted diet but growing well; I would be comfortable with <120umol/L in maternal PKU where I was certain formula and calorie intake was optimized and the patient was careful with foods they chose to ensure good nutrition; If patients are experiencing rapid growth (usually in infancy); Only for super responders to Kuvan tolerating DRI total protein from regular protein foods with minimal or no PKU foods”

Blood Phe and tyrosine were reported as being monitored by all dietitians, with 95% also monitoring ferritin (data not shown). More than half also routinely monitor pre-albumin, albumin and vitamins. Forty-seven percent reported routinely monitoring bone density while a small minority reported routine monitoring of essential fatty acids. Among other routinely monitored surrogate biomarkers, homocysteine, carnitine, full amino acid quantification, alkaline phosphatase, complete blood count, trace elements (zinc, selenium, manganese), folate, B12, and 25-hydroxyvitamin D were reported by some respondents (data not shown).

For younger patients, all respondents indicated that the target range for blood Phe levels was 120–360 μmol/dL, but opinions varied slightly for patients aged > 10–18 years old: most dietitians recommended 120–360 μmol/L, while some recommended higher target Phe levels, up to 600 μmol/L (Table 3). The majority of respondents consider 120 μmol/L to be the lowest acceptable average level for blood Phe, in the long-term (Table 3). A majority would rarely recommend keeping blood Phe levels at the lower end of therapeutic range, by means of a more phe-restricted diet, and specifically would not be comfortable with patients having blood Phe levels lower than 120 μmol/L (Table 3). Nearly half of the respondents (47%) recommend maintaining blood Phe levels at a higher-end of the therapeutic range for “some patients” (clinical case scenarios were not specified) (Table 3).

### Clinic visits and team communication

As expected, clinic visits were most frequent in infants 0–12 months old, and declined in older age groups (Table 4). After the first year of life, the majority of dietitians indicated seeing their patients less often than once per month, but at least once per year. Similarly, a majority of respondents reported that between-visit communications took place most often with parents of the youngest patients (Table 4). With respect to the means of communication with families between visits, the telephone was used by more dietitians (100%), than was email (89%), mail (58%), fax (32%), and phone texts (16%) (data not shown).

**Table 4:** Clinic visits and communication

	At least once per week	Less than once per week but at least once per month	Less than once per month but at least once per year	Less than once per year	Other	GMDI guideline^a^
					n (%)	
						n (%)
				n (%)		
	n (%)					
		n (%)	n (%)			
Reported frequency of clinic visits^b^						
< 1 year of age	3 (17)	6 (33)	8 (44)	0	1 (6)	weekly to monthly
1–2 years of age	0	4 (22)	14 (78)	0	0	monthly to every 6 months
3–10 years of age	0	2 (11)	16 (89)	0	0	
11–18 years of age	0	2 (11)	16 (89)	0	0	each 6–12 months
Reported frequency of communication with patients/family^c^						
< 1 year of age	15 (79)	3 (16)	0	0	1 (5)^d^	1–2 times per week
1–2 years of age	4 (22) ^b^	13 (72) ^b^	0	0	1 (6)^b,e^	once per week to once per month
3–10 years of age	0	13 (69)	5 (26)	0	1 (5)^e^	
11–18 years of age	0	9 (47)	9 (47)	0	1 (6)^f^	

^a^GMDI recommendations are based on the following age groups: 0–1 year, 1–7 years; 8–18 years

^b^One missing response (*n* = 18)

^c^via phone, email, fax, and other means of communication outside of the clinic visit

^d^“Each month until 3 months old then every 3 months”

^e^“Based on how frequently family/patients monitor Phe levels. Some [monitor] weekly, some [monitor] every 2 weeks, some [monitor] monthly”

^f^“Blood work is supposed to be done monthly; if they do it, I will connect with them”

All respondents reported discussing individual patients’ nutritional management with other members of the health care team. However, only slightly more than half (11/19, 58%) indicated discussing most of their patients on a regular basis, and under a half of respondents (8/19, 42%) reported that these discussions do not occur routinely. Just over one quarter (5/19, 26%) consider multidisciplinary healthcare team communication to be “highly effective”, while the majority of respondents (13/19, 68%) report that they find within-team communication to be “somewhat effective”, and one dietitian considers it to be not effective (1/19, 6%).

### Dietary prescription and assessment

The most important factors reported to influence the prescription for medical food (formula) were the nutrient composition of formula, patient’s age, preferences of the patient or family and availability of the product, reported by 95, 89, 89 and 79% of dietitians, respectively (Table 5). The most commonly prescribed formulas (proportion of dietitians including the formula as within their “top 3”) were: Periflex Infant (53%) and Phenyl free 1 (37%) for infants < 1 year old; Phenyl free 1 (26%) and Periflex Junior (26%) for 1–2 year-olds; Periflex Junior (21%) and Periflex Junior Plus (21%) for children aged 3–9 years; and Periflex Advance (21%) and Phenylade Essential (21%) for children aged 10–18 years (some of the responses with regard to the different Periflex products reflect periods of transition in their availability). One third of participants (32%) reported that their choice of formula is limited by the hospital formula contract. Full provincial coverage of the costs of low protein foods was reported by dietitians from 4 centres, while the remainder reported only partial coverage.

**Table 5:** Diet prescription, assessment, and monitoring

	n (%)
Factors considered “very important” in deciding which formula to prescribe^a^	
Nutrient composition of formula	18 (95)
Patient’s age	17 (89)
Preferences of the patient or family	17 (89)
Availability of the product	15 (79)
Price of the product	4 (21)
Severity of PAH deficiency	4 (21)
Approaches to assessing patient adherence to formula and diet prescription^a^	
By patient’s/caregiver’s verbal report	17 (89)
Monitoring weight and height	15 (79)
By laboratory tests:	
Phenylalanine	16 (84)
Pre-albumin	13 (68)
Tyrosine	13 (68)
Iron	12 (63)
B12	12 (63)
Albumin	5 (26)
Checking how much formula was released by the dispensing authority	12 (63)
By analyzing written dietary questionnaires	10 (53)
Technique that is *most* successful in improving adherence to diet	
Individualized PAH deficiency nutrition counseling	11 (58%)
Motivational interview techniques	4 (21%)
Reporting results of blood dots to patients	4 (21%)
Regular reminders for Phe blood dots	0
Nutrients that are routinely monitored for “most” patients, based on diet records^a^	
Dietary Phe intake	19 (100%)
Protein intake	18 (95%)
Calorie intake	14 (74%)
Mineral intake (any)	12^b^ (67%)
Vitamin intake (any)	12 (63%)
Fat intake	5^c^ (29%)
Other	0
Components that are always/often included in routine clinical visit assessments^a^	
Anthropometric measurements	19 (100)
Diet education	17 (90)
Dietary analysis	17 (90)

^a^Multiple choice questions, the percentages are expected to add up to more than 100%

^b^One missing response (*n =* 18)

^c^Two missing responses (*n =* 17)

The discontinuation of medical formula was reported as “never” considered by 8/19 (42%) of respondents, while 11/19 (58%) respondents would consider discontinuing formula in some cases; for example, patients with mild PAH deficiency and those who are good responders to Kuvan (sapropterin dihydrochloride, BH4) (data not shown). With regard to low protein food, good and excellent accessibility was reported by the majority of responders (17/19, 89%). A minority of dietitians (4/19, 21%) reported prescribing large amino-acid supplements (LNAAs) to their pediatric patients.

A majority of dietitians (17/19, 89%) reported 3-day diet records as most frequently used for monitoring nutrition intake adequacy. “Records for the 2 days prior to the bloodwork” and “2 day diet record” were mentioned by two respondents (2/19, 11%) (data not shown). The most frequently recommended method for self-monitoring of Phenylalanine intake was counting grams of dietary natural protein, reported by (17/19, 89%), followed by regular bloodwork (14/19,74%), counting milligrams of dietary Phenylalanine (12/19, 63%), counting Phe exchanges (9/19, 47%) and use of computer applications for PKU (9/19, 53%). Out of those, “counting milligrams of dietary phenylalanine” was the most often recommended method for self-monitoring intake of phenylalanine (7/19, 37%), followed by “counting grams of dietary natural protein” (4/19, 21%). Those who use computer applications, indicated “How much Phe?” as the most frequently reported application (67%), followed by “Accugo” (25%) and “Metabolic Diet App” (25%). Those who reported using Phe exchanges (9/19,47%), reported calculating 1 exchange as 15 mg of Phenylalanine.

Respondents most frequently reported home sample collection as the method of collecting blood samples for routine monitoring of phenylalanine (95%), followed by a “local lab or hospital close to patient’s house” (68%) and “metabolic clinic” (63%) (data not shown).

### Monitoring adherence to the medical formula and low protein foods

To assess patients’ adherence to formula intake, dietitians most often reported relying on the verbal report of the parent and/or caregiver (89%), followed by monitoring blood Phe levels (84%), monitoring weight and height (79%), checking how much formula was released by the dispensing authority (63%) and analyzing written dietary questionnaire (53%) (Table 5). As expected, a majority of respondents consider high blood Phe levels to be the most reliable indicator of patients’ non-adherence to the diet and/or drug therapy (10/19, 53%), followed by “not pulling formula from the sources that supply formula” (5/19, 26%), “not doing blood dots on a regular basis” (3/19, 16%) and “not showing up in clinic” (1/19, 5%) (data not shown).

To improve a patient’s adherence to the diet, dietitians employ several strategies, including individualized nutrition counseling (reported by19/19, 100%), motivational interview techniques and reporting results of blood Phe dots to patients (14/19, 74% and 14/19, 74% respectively), and regular reminders to collect/submit blood Phe dots (10/19, 53%). However, regular reminders to collect/submit blood Phe dots were reported to be the least successful of the strategies (Table 5).

Intake of dietary Phe, protein, calories, minerals, and vitamins are routinely monitored for most patients, as reported by the majority of participants (Table 5). All participants reported performing anthropometric measurements at every clinic visit; while both diet analysis and nutrition education were reported as always/often included in routine visits by 90% of respondents (Table 5).

## Discussion

### Reported use of guidelines

All respondents were aware of the ACMG and GMDI PAH deficiency consensus guidelines, and almost all respondents reported use of these guidelines. With respect to the GMDI nutrition guidelines, in particular, more detailed information and discussion is provided online at the SERN-GMDI PKU Nutrition Guidelines website including a PKU tool kit with detailed patient diet examples for dietitians [21]. The guidelines are in widespread use but, given the lack of evidence, they often do not recommend a specific course of action related to the most uncertain clinical practice questions (e.g., diet initiation in mild PAH deficiency). These areas of uncertainty were among the most variable aspects of nutritional management reported by the Canadian dietitians in our survey.

### Human resources and services in metabolic centres

Consistent with a previous report [24], our survey identified variation in the organization of care within Canadian metabolic centres. Although the evidence with respect to the impact of a coordinated team approach on improved outcomes in the treatment of PAH deficiency is very scarce, one Canadian retrospective study reported that a multidisciplinary centralized approach results in better outcomes in terms of improved adherence to the diet, control of blood Phe, and fewer patients lost to follow up [13]. Both recent American and European guidelines recommend a multidisciplinary coordinated approach to the management of PAH deficiency, where the health care team should include a metabolic physician, dietitian, specialized metabolic laboratory and access to a psychologist and a social worker. Our survey indicated that only 3 out of 14 centres have a metabolic physician, dietitian, biochemist and access to a psychologist; indicating a lack of multidisciplinary care. Only two centres reported having dietitians whose time is fully dedicated to the care of patients with PAH deficiency, but seven centres reported at least a half time dedicated position. These differences likely reflect patient numbers but may also reflect differences in staff time available for patient care. With regard to the communication within the healthcare team, only one quarter of survey respondents regarded this as highly effective, highlighting a need to improve existing communication practices within healthcare teams who provide care to patients with PAH deficiency.

### PAH deficiency phenotype classification

Our survey revealed limited consensus among Canadian dietitians on the definition of the severity of PAH deficiency. To identify the type of PAH deficiency, the majority of dietitians reported use of pre-treatment blood Phe levels alone, or in combination with Phe tolerance and/or genotype. A few dietitians either do not use pre-treatment blood Phe levels for this purpose, or else use them for a modified classification, such as “HPA or classical PKU”. Such a lack of clarity most likely created a discrepancy in reporting the use of pre-treatment blood Phe levels, to define the severity of PAH deficiency: Fig. 1 indicated that only two dietitians do not use pre-treatment blood Phe levels for this purpose, but the number increased to four in Table 2, in response to a request to provide a range for each classification of PAH deficiency (PKU); mild HPA, mild PKU, moderate PKU, classical PKU.

Several authors have recommended against each of the indicators as a means of classifying disease severity in the neonatal period [1, 25]. For example, pre-treatment blood Phe levels typically do not reach a maximum because of prompt diagnosis and treatment onset [1]. In addition, precise Phe tolerance is difficult to determine at the clinic visit setting because of inconsistencies between actual and prescribed dietary Phe intake and other factors, such as a patient’s age and/or metabolic state during the period of interest [25]. Finally, PAH genotypes are often difficult to interpret because several mutations are responsible for wide range of clinical phenotypes [3, 26]. Since none of the above criteria are fully appropriate as a standard for the classification of PAH deficiency, the most recent north American guideline^3^ referred to a previous NIH consensus guideline^16^ that suggested a simplified classification which is based on pre-treatment blood Phe levels [27]. Therefore the respondents to this survey were generally following established practice.

Determining the severity of PAH deficiency might not seem crucial in the clinical setting where a patient’s management is rather dynamic, and directed by the most current blood Phe levels. However, there is a small but feasible risk that overestimation of the degree of severity of PAH deficiency could initially result in over-restriction of the intake of natural protein, until Phe tolerance is empirically determined. Furthermore, if an individual is assumed to have minimal residual PAH activity, and therefore potentially a low chance of responding to sapropterin, he or she may also not be given the opportunity for a BH4 responsiveness trial [28, 29].

### Frequency of clinic visits and provider-family communication

Individual adherence to nutrition therapy depends on numerous patient- and healthcare-related factors, and appears to decline with increasing patient age [10, 30]. There is some evidence that gaps in communication between health care providers and patients / families may contribute to nonadherence [11]. As recommended by the 2014 treatment guidelines for PAH deficiency from GMDI, frequency of communication with patients, aged 8–18 years, should occur weekly to monthly [2]. However, nearly half of the survey respondents reported communicating with patients of this age and their families, less frequently than is recommended. Aligned with recommendations though, was the contact with 3–10 year old patients and their families: 68% of survey respondents reported their frequency of communication to be 1–3 times per month. Decreased frequency of contact with older children is likely due to decreased frequency of home blood Phe monitoring, especially as patients learn to become independent in managing their daily diets and home blood draws. However, other factors may also offer an explanation for the failure to meet recommendations: staff shortages in the metabolic clinic and subsequent time limitations; disappointment with non-adherent patients; other social, psychological, economical and human resource related barriers [31–33]. The decline in the frequency of communication that we observed might contribute to non-adherence with treatment in adolescents. The evidence suggests that continuing communication and education throughout childhood, and perhaps reinforcement of the frequency and quality of communication might promote better adherence and subsequently may improve long-term outcomes in older children and adults [11].

We did not ask participants about the frequency of blood Phe measurements. However, we believe that there is a relatively close correspondence between the frequency of communication of dietitians with patients/families and the frequency of blood Phe measurements, since typically each blood Phe result triggers communication with the patient / family.

### Treatment initiation

There is a good evidence and expert agreement that treatment for PAH deficiency should be initiated at ≥600 μmol/L (10 mg/dL) [3, 34]. However there is lack of conclusive evidence on the balance between “added benefit” and “no harm” of treatment initiation at ≥360–600 μmol/L (6–10 mg/dL). This uncertainty translates into provisional practice recommendations [3, 12, 34]. Not surprisingly, our survey found that the majority of dietitians set the threshold for initiation of therapy at ≥360 μmol/L (≥6 mg/dL), and several others at higher blood Phe levels. In alignment with the evidence and published guidelines, all would begin dietary Phe restriction when the blood Phe level is ≥600 μmol/L (10 mg/dL).

### Prescribing LNAA

Since this was a survey of pediatric practice, less than a quarter of respondents reported prescribing supplements of large neutral amino acids (LNAA). Animal and human studies show that Phe competes with LNAAs for the protein carrier through the intestinal wall and the blood brain barrier. Thus the lack of LNAAs, in of itself, might promote higher Phe levels in the central nervous system [35, 36]. There is mainly positive but limited evidence on the benefit of LNAA supplementation in treatment of PAH deficiency. Therefore, as the LNAA content in PKU medical foods (formulas) can vary, more research especially on the safety and long-term outcomes of treatment with LNAAs is clearly needed [37, 38]. As mentioned in the ACMG guidelines, current use of LNAA is limited to adolescents and adults, with avoidance in pregnancy. A European panel of PKU experts gave no statement on the use of LNAAs [3, 14].

## Limitations

Our survey focused on PAH deficiency in the pediatric population and thus we cannot comment on the transition to adult care, nor to adult nutrition management.

While our response rate was reasonable (59%) for a survey of health care providers and represented almost all Canadian metabolic centers (14 out of 16) and provinces and territories, with the exception of Nunavut and Newfoundland and Labrador, the views of participants may not represent those of all metabolic dietitians in Canada; for example, the individuals who did not respond to the survey may be less aware of, or adherent to, current guidelines. Another major limitation of this survey was that we did not address how much Phe, tyrosine and protein (medical foods/formulas and natural protein), in relation to age, was prescribed in each center; nor what proportion of these prescriptions were aligned with recommendations. We believed that such detailed nutritional data should be derived from clinical reviews (e.g., from chart reviews), which was outside the scope of this publication.

## Conclusion

We found that Canadian metabolic dietitians generally follow published guidelines in their nutritional management of pediatric PAH deficiency. Dietitians responded with some variation, both across and within centres. The most striking differences were in approaches to defining PAH deficiency phenotype, treatment targets for blood Phe levels, frequency of clinic-patient communication with older children, and organization of care in metabolic centres. More research is needed to generate better evidence, with which to address the current gaps in knowledge in relation to treatment of PAH deficiency, variation in laboratory monitoring and clinic visit frequency; with subsequent translation into practice.

## Abbreviations

ACMG: American College of Medical Genetics and Genomics
CIMDRN: Canadian Inherited Metabolic Diseases Research Network
GMDI: Genetic Metabolic Dietitians International
HPA: Hyperphenylalaninemia
IMD: Inherited metabolic diseases
NIH: National Institutes of Health
OMIM: Online mendelian inheritance in man
PAH: Phenylalanine hydroxylase
Phe: Phenylalanine
PKU: Phenylketonuria
REDCap: Research electronic data capture
SERN: Southeast regional genetics network
